# Supplementary material for: Analysis of chi angle distributions in free amino acids via multiplet fitting of proton scalar couplings
Source: Magn Reson (Gott). 2024 Aug 19;5(2):103–20. doi: 10.5194/mr-5-103-2024 (PMC11570886; doi:10.5194/mr-5-103-2024)
Supplement: The supplement related to this article is available online at: https://doi.org/10.5194/mr-5-103-2024-supplement. [file mr-5-103-2024-supplement.zip › Syed2024/data/fit1d_gissmo.tar.gz!/fit1d/pro/500/bmse000047_simulation_1_nmredata.zip!/bmse000047_simulation_1_nmredata/1H/index.html_C=S_O=D]

Index of /ftp/pub/bmrb/metabolomics/standards/L\_proline/nmr/bmse000047/1H


# Index of /ftp/pub/bmrb/metabolomics/standards/L\_proline/nmr/bmse000047/1H

|  | Name | Last modified | Size | Description |
| --- | --- | --- | --- | --- |
| --- | | | | |
|  | Parent Directory |  | - |  |
|  | fid | 14-Jan-2011 15:39 | 77K |  |
|  | uxnmr.par | 14-Jan-2011 15:39 | 17K |  |
|  | acqus | 14-Jan-2011 15:39 | 9.0K |  |
|  | acqu | 14-Jan-2011 15:39 | 8.8K |  |
|  | prosol\_History | 14-Jan-2011 15:39 | 5.9K |  |
|  | shimvalues | 14-Jan-2011 15:39 | 2.9K |  |
|  | format.temp | 14-Jan-2011 15:39 | 2.7K |  |
|  | pulseprogram | 14-Jan-2011 15:39 | 2.1K |  |
|  | audita.txt | 14-Jan-2011 15:39 | 886 |  |
|  | scon2 | 14-Jan-2011 15:39 | 462 |  |
|  | pdata/ | 14-Jan-2011 15:39 | - |  |
| --- | | | | |

Apache/2.2.3 (CentOS) Server at www.bmrb.wisc.edu Port 80
